# Intestinal *Mycobacterium avium* complex infection: a rare case of small-bowel atrophy

**DOI:** 10.1055/a-2388-7169

**Published:** 2024-09-04

**Authors:** Michael Visentin, Lucia Scaramella, Alessandra Bandera, Marco Maggioni, Luca Elli

**Affiliations:** 1 9339 Gastroenterology and Endoscopy Unit, Fondazione IRCCS Caʼ Granda Ospedale Maggiore Policlinico, Milan, Italy; 2 Department of Pathophysiology and Transplantation, University of Milan, Milan, Italy; 3 9339 Infectious Diseases Unit, Fondazione IRCCS Ca' Granda Ospedale Maggiore Policlinico, Milan, Italy; 4 9339 Division of Pathology, Fondazione IRCCS Ca’ Granda Ospedale Maggiore Policlinico, Milan, Italy

We report the case of a 57-year-old woman with acquired immunodeficiency syndrome (AIDS) who was admitted to the emergency room with fever, diarrhea, and severe malnutrition (body mass index [BMI] 15.8 kg/m ^2^ ). Her history was notable for human immunodeficiency virus (HIV) infection with poor therapeutic adherence, which had been complicated by multiple opportunistic infections. Esophagogastroduodenoscopy and colonoscopy were macroscopically normal. A video capsule endoscopy was performed, which revealed diffuse jejunal atrophy, and whitish and edematous enteric mucosa with scalloping ( Video 1 ). Subsequently, anterograde double-balloon enteroscopy confirmed significant signs of atrophy with scalloping and a mosaic pattern in the jejunum ( Fig. 1 ). Subsequent histologic examination raised the suspicion of *Mycobacterium avium* complex (MAC) ( Fig. 2 ), which was confirmed afterward by polymerase chain reaction (PCR). Treatment was therefore initiated with rifabutin, azithromycin, and ethambutol with clinical improvement.

**Fig. 1:**
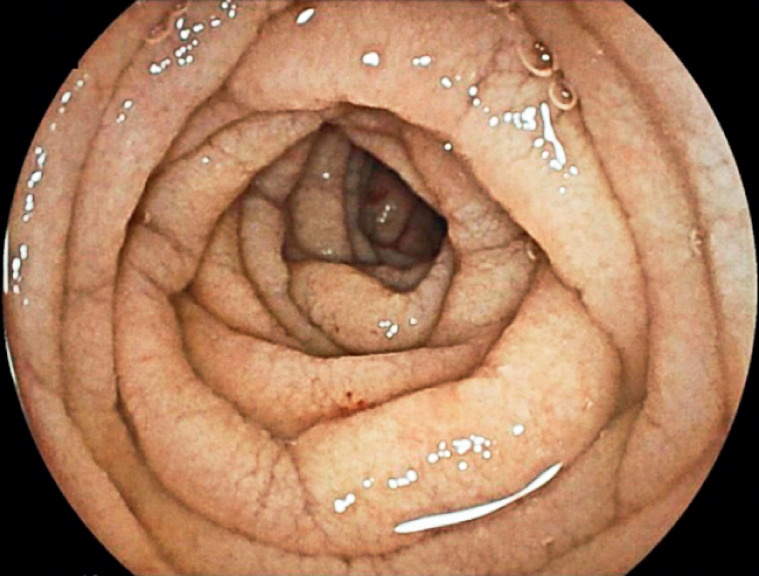
Image during anterograde double-balloon enteroscopy showing signs of atrophy, scalloping, and a mosaic pattern of the jejunum in a patient with *Mycobacterium avium* complex disease.

**Fig. 2:**
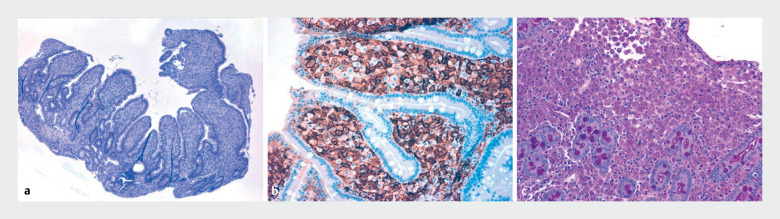
Histologic appearance showing: **a** ileal mucosa with diffusely enlarged villi (hematoxylin and eosin [H&E] stained; magnification × 40); **b** ileal lamina propria filled with histiocytes (CD68 immunostaining; × 200); **c** histiocytic cytoplasm full of periodic acid–Shiff (PAS)-positive bacilli (PAS stained; × 200).


Video capsule endoscopy showing diffuse jejunal atrophy, and whitish and edematous enteric mucosa with scalloping in a patient with *Mycobacterium avium* complex disease.
Video 1

Disseminated MAC is an infection caused by a nontuberculous mycobacterial species 1 , with this type usually associated with HIV infection; however, the widespread use of effective antiretroviral therapy and the use of prophylaxis against MAC infection have reduced the incidence of this illness 2 . This case describes a rare manifestation of an infrequent opportunistic infection that is typical of AIDS patients. In addition, we report detailed imaging and video documentation of a MAC-driven enteropathy to support endoscopists and clinicians in their everyday practice.

Endoscopy_UCTN_Code_CCL_1AB_2AH_3AB

## Notes

Endoscopy E-Videos https://eref.thieme.de/e-videos
*E-Videos* is an open access online section of the journal *Endoscopy* , reporting on interesting cases and new techniques in gastroenterological endoscopy. All papers include a high-quality video and are published with a Creative Commons CC-BY license. Endoscopy E-Videos qualify for HINARI discounts and waivers and eligibility is automatically checked during the submission process. We grant 100% waivers to articles whose corresponding authors are based in Group A countries and 50% waivers to those who are based in Group B countries as classified by Research4Life (see: https://www.research4life.org/access/eligibility/ ). This section has its own submission website at https://mc.manuscriptcentral.com/e-videos .

## References

[1] Grinsztejn B Fandinho FC Veloso VG Mycobacteremia in patients with the acquired immunodeficiency syndrome Arch Intern Med 1997 157 2359 9361577

[2] Palella FJ Jr Delaney KM Moorman AC Declining morbidity and mortality among patients with advanced human immunodeficiency virus infection. HIV Outpatient Study Investigators NEJM 1998 338 853 9516219 10.1056/NEJM199803263381301

